# Extreme heat changes post-heat wave community reassembly

**DOI:** 10.1002/ece3.1490

**Published:** 2015-05-06

**Authors:** Linda I Seifert, Guntram Weithoff, Matthijs Vos

**Affiliations:** 1 Department of Ecology and Ecosystem Modelling, Potsdam UniversityMaulbeerallee 2, 14469, Potsdam, Germany; 2 Working Group Zoological Biodiversity, Ruhr-Universität BochumUniversitätsstr. 150,Gebäude ND05, D-44780, Bochum, Germany; 3 Department of Conservation Biology, Institute of Environmental Sciences (CML), Leiden UniversityPO Box 9518, 2300, RA Leiden, The Netherlands

**Keywords:** Biodiversity, climate change, conservation, ecological restoration, extinction, extreme temperature events, global warming, maximum temperature, variability

## Abstract

Climate forecasts project further increases in extremely high-temperature events. These present threats to biodiversity, as they promote population declines and local species extinctions. This implies that ecological communities will need to rely more strongly on recovery processes, such as recolonization from a meta-community context. It is poorly understood how differences in extreme event intensity change the outcome of subsequent community reassembly and if such extremes modify the biotic environment in ways that would prevent the successful re-establishment of lost species. We studied replicated aquatic communities consisting of algae and herbivorous rotifers in a design that involved a control and two different heat wave intensity treatments (29°C and 39°C). Animal species that suffered heat-induced extinction were subsequently re-introduced at the same time and density, in each of the two treatments. The 39°C treatment led to community closure in all replicates, meaning that a previously successful herbivore species could not re-establish itself in the postheat wave community. In contrast, such closure never occurred after a 29°C event. Heat wave intensity determined the number of herbivore extinctions and strongly affected algal relative abundances. Re-introduced herbivore species were thus confronted with significantly different food environments. This ecological legacy generated by heat wave intensity led to differences in the failure or success of herbivore species re-introductions. Reassembly was significantly more variable, and hence less predictable, after an extreme heat wave, and was more canalized after a moderate one. Our results pertain to relatively simple communities, but they suggest that ecological legacies introduced by extremely high-temperature events may change subsequent ecological recovery and even prevent the successful re-establishment of lost species. Knowing the processes promoting and preventing ecological recovery is crucial to the success of species re-introduction programs and to our ability to restore ecosystems damaged by environmental extremes.

## Introduction

Under climate change major regions on the planet will experience both gradual warming and an increase in temperature variability. In the past three decades, such variability has already emerged in the form summertime extremely hot outliers (Hansen et al. 2012). In the coming decades, hot extremes are expected to occur with increasing intensity, duration and frequency, in many locations, on a considerable fraction of the planet's surface (Karl and Trenberth 2003; IPCC 2007; Quesada et al. 2012; Fischer et al. 2013). Variability in terms of environmental extremes poses a greater threat to species and biodiversity than slow and gradual warming itself (Vasseur et al. 2014). Accordingly, research has started to not only focus on gradual increases in mean temperatures (see Bale et al. 2002; Brown et al. 2004) but also on the effects of increasingly severe extreme events (Jentsch et al. 2007) such as heat waves (e.g., Sentis et al. 2013) and other catastrophic climatic events such as floods (Thibault and Brown 2008), droughts (Mueller et al. 2005; Bogan and Lytle 2011) and storms (Pringle and Hamazaki 1997; Batista and Platt 2003).

Some of the ecological consequences of extreme events are straightforward. Environmental extremes that exceed the tolerance limits of many species in a region enhance mortality and are likely to promote community-wide population declines that may result in local extinctions. This implies that ecological systems may need to increasingly rely on recovery via community reassembly assisted by meta-community dynamics, that is, by dispersal-mediated recolonization.

However, how community reassembly will proceed is not easy to predict. Historical effects, such as the order in which species go extinct and are re-introduced, can be essential for the emerging patterns (Fukami et al. 2010). Ecological legacies may also be generated in other ways. For example, an extreme in the abiotic environment may not only drive a species locally extinct (by a breach of tolerance limits), but may also change the composition of the remaining community, that is, the biotic conditions, in ways that change the ability of that species to exist within it later on, when the abiotic conditions have returned to normal. Any process that creates such historical effects or ecological legacies may affect the outcome of community assembly or reassembly. To what extent extreme events of different intensities differ in their propensity to create alternative ecological legacies, which affect postevent recovery, is largely unknown.

On the one hand, strong ecological forces may have canalizing effects that lead to rather reliable successional patterns and recovery (Pearson and Rosenberg 1978; Berlow 1997), but on the other hand, noise-enhancing mechanisms, alternative attractors, and historical contingencies may lead to severe unpredictability and a wide range of possible transients and outcomes of the recovery process (Drake 1990, 1991; Berlow 1997; Fukami et al. 2010; Fukami and Nakajima 2011). It is presently not clear whether extreme events of different intensities tend to “reset” ecological succession in a similar way or will tend to create different ecological legacies that change subsequent recovery.

It can, however, be expected that higher intensity extremes lead to higher mortality, stronger population declines, and hence to more frequent local extinctions. This “extraction” of ecological players could, in itself, create more room and degrees of freedom for noise enhancement. This in turn would result in higher postextreme community-level variability than would be the case following a lower intensity extreme event that caused fewer extinctions.

Here, we focus on the consequences of differences in heat wave intensity for the dynamics, species loss, and reassembly of ectotherm communities. Ectotherms comprise the majority of all animal species on the planet and will be rather directly affected by extreme heat events as their metabolism, feeding rate, and overall activity is largely determined by ambient temperature (Wilson 1992; Deutsch et al. 2008). Very high temperature negatively affects ectotherms because it causes respiration to outpace resource intake, leading to a net loss of energy and to enhanced mortality (Walz 1993; Seifert et al. 2014, in press^2015^). An increasing intensity and frequency of heat waves may thus cause ectotherm population declines and lead to a higher rate of local species extinctions. This would lead to associated changes in the balance of competition and predation, which could prevent the re-establishment of some of the lost species, even if they were previously viable and successful as residents. Lundberg et al. (2000) appropriately coined the term “community closure” for this phenomenon. Community closure could prove to be a serious problem in a world experiencing a regime of increased environmental extremes as it could prevent successful ecosystem recovery through the “usual mechanism” of dispersal-mediated re-colonization, that is, through meta-community dynamics and the associated spatial insurance (Loreau et al. 2003). It is presently unknown whether extreme event intensity itself increases the likelihood of community closure.

To study the effects of differences in heat wave intensity on subsequent community recovery, we tested the following hypotheses: (H1) Differences in heat wave intensity lead to differences in community dynamics including numbers of extinctions and relative abundances of the remaining species. As a consequence, (H2) differences in heat wave intensity then also lead to differences in the likelihood of community closure during the process of community reassembly. Finally, we hypothesize (H3) that a higher heat wave intensity, that is, more extreme heat, leads to a more variable outcome of the community reassembly process.

## Methods

### Model community

The inoculated herbivore community consisted of five rotifer species; *Brachionus calyciflorus* (origin: Lake Michigan, USA) *B. havanaensis* (origin: Mexico City, Mexico), *Keratella quadrata* (origin: Potsdam, Germany), *Cephalodella* sp. (origin: New Jersey, USA, see Altermatt et al. (2011)), *Lecane* sp. (origin: Potsdam, Germany), two green algae: *Monoraphidium minutum* (243-1, SAG Göttingen, Germany) and *Nannochloropsis limnetica* (18.99, SAG, Göttingen, Germany) and two flagellates (*Chlamydomonas* and a Chrysophyte). We based herbivore inoculation densities on carbon content. *B. calyciflorus* and *B. havanaensis* individuals contain about 0.05 and 0.03 *μ*g carbon; the value for *Keratella* was estimated at 0.02 *μ*g carbon (Telesh et al. 1998) and for *Cephalodella* and *Lecane* both at 0.0125 *μ*g carbon. For community assembly, we used the following initial densities: *B. calyciflorus* (1250 ind L^−1^)*, B. havanaensis* (2500 ind L^−1^)*, Keratella* (3750 ind L^−1^), *Cephalodella* (5000 ind L^−1^), *Lecane* (5000 ind L^−1^), representing similar densities in carbon. Initial densities of the main food algae were 1.5 mg C L^−1^ *Monoraphidium* and 1.5 mg C L^−1^ *Nannochloropsis*. In addition, we introduced small densities (<0.1 mg C L^−1^) of two flagellates, *Chlamydomonas* and a Chrysophyte, to all replicates. We used four different phytoplankton species (rather than a single resource) in the expectation that this would allow coexistence and support viable population growth for at least four of the herbivore species. Hereafter, we present all densities of algae and rotifers in terms of biomass (mg C L^−1^) rather than in numbers of individuals, for easy comparison of densities at different trophic levels.

Stock cultures were maintained at 20°C in WC medium (Guillard and Lorenzen 1972). Temperature was decreased by 1°C per 12 h until the experimental starting temperature of 15°C was reached; the acclimation period lasted 1 week. Acclimation and experiments occurred in climate controlled chambers (Minitron, INFORS HT, Bottmingen, Switzerland) in continuous light (50 ± 10 *μ*mol photons (PAR) m^−2^ sec^−1^). Cultures were kept in suspension by gentle shaking (60 rpm).

### Experiments

We exposed model communities to one of two different heat wave intensities. The moderate heat wave treatment was warmed from 15°C to 29°C, whereas the extreme heat wave treatment was warmed to 39°C. Twenty-nine degree Celsius represents a high summer heat wave temperature for a temperate system, whereas 39°C represents about the present planetary daily maximum temperature of a tropical aquatic system, such as a very shallow reservoir, pond, pool, lake, or lagoon, and is known to lead to a dis-balance of respiration and food intake in herbivorous ectotherm rotifers such as *Brachionus calyciflorus* (Galkovskaja 1987). In both these ways, 39°C represents a boundary value and this temperature was expected to definitely induce herbivore species extinctions. Note that extremely hot outliers (a new category of very hot heat waves of an intensity that did not exist in the decades before the 1980s) are expected to increase in frequency and intensity, for a considerable part of the planet's surface (Hansen et al. 2012).

Experiments ran in 200 mL microcosms at 15°C, for an initial period that lasted from day 1 to day 7. We defined viable community members as all those species whose population density showed positive growth during this initial 1-week period. On basis of pilot experiments, we decided to regard any species showing a major decline during this 1-week period (i.e., even before the heat wave) as marginal species most likely heading for extinction. All species, except *Keratella*, showed positive growth during the initial 1-week period. Heat waves started on day 8, when temperature was increased within 15 h with a continuous stepwise increase of 2.8°C/3 h to 29°C or 4.8°C/3 h to 39°C. Temperature was then kept at these levels for 24 h (day 9) and cooled down through a reversal of the original stepwise increase at day 10 within 15 h to the initial level (15°C). Heat waves were assumed, on basis of pilot experiments, to induce species extinctions. The course of community development was followed by sampling 11% of the volume every other day, and replacing this volume with fresh medium. As a proxy for extinction, we used the criterion that a species was not detectable for 2 or more subsequent samplings. On day 16 (after sampling and counting), we re-introduced all viable species that had been lost after the heat wave into the community at a density of one-fifth of their initial density, that is, 250, 500, and 1000 ind L^−1^ for *B. calyciflorus*, *B. havanaensis,* and *Lecane*, respectively. Replicates that did not receive a heat wave served as controls. Experiments ran in replicate (*n* = 6) for each treatment and for the controls, leading to a total of 18 experimental communities. As both algae and the rotifers reproduce within days, this was a multigeneration community experiment. Rotifers were counted by eye, using the entire 20 mL sample and an inverted microscope (10–40x), except for *Cephalodella* that was counted using subsamples of 5 mL on days 5–8 for all treatments and using 1 mL on days 10–30 for controls and heat treatments, due to its high abundance. Algal densities were determined with an inverted microscope (40x) using the cell counting method (Peters 1984). At least, 600 algal cells were counted for each sample. Algae were counted for days 8, 10 (the start and end of heat waves), 16 (the day of re-introduction), and 30 (the end of the experiment).

### Statistical analysis

To analyze differences in herbivore community dynamics (in relation to Hypothesis 1), we employed a repeated-measures MANOVA using the general linear model function of SPSS.

Per replicate, we averaged the densities for the different sampling dates, for each of the three periods (that were of different lengths): the period before the heat wave, the subsequent period from when the heat wave is applied until the species re-introduction(s), and then the period following the species re-introduction(s). These data were log(*x* + 1)-transformed to deal with heterogeneity of variances. As Mauchly's test showed sphericity was (still) violated, we applied a Greenhouse–Geisser correction of the degrees of freedom.

To test for differences in the number of extinctions (H1) and in the occurrence of community closure between the two heat wave treatments (H2), we simply used a Mann–Whitney *U*-test.

To analyze variability in the eventual outcome of community reassembly (in relation to Hypothesis 3), we used the average biomass within each replicate taken over the last three sampling dates (day 26, 28, and 30). We then measured the variability among replicates within both “moderate” and “extreme” treatments using the Morisita similarity index, which is recommended as it is not sensitive to diversity and sample size (Wolda 1981): 
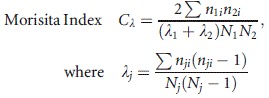
*n*_*ji*_* *= the number of individuals of species *i* in sample *j*; *N*_*j*_* *= the number of individuals in sample *j*.

The Morisita index has a maximum value for similarity that can be a bit higher than 1 (Wolda 1981). Values close to zero indicate dissimilarity whereas values close to 1 indicate a high similarity. We tested for differences in Morisita index values between the post-29°C heat wave community and the post-39°C heat wave community treatments using a Mann–Whitney *U*-test. This test compared 15 index values (based on all possible pairs of the six replicates) of the 29°C treatment with the 15 index values for the 39°C treatment. As these pairs are not independent of each other, we chose to apply a more stringent critical level of 0.05/15 = 0.0033.

To visualize similarities/dissimilarities among replicates and between treatments, we applied multidimensional scaling using MatLab (The MathWorks, Inc., cmdscale-protocol). For the MDS plot, we used untransformed mean abundance data for each species for the entire post-re-introduction period.

## Results

### Effects of heat wave intensity on community dynamics and extinctions (H1)

**Figure 1:**
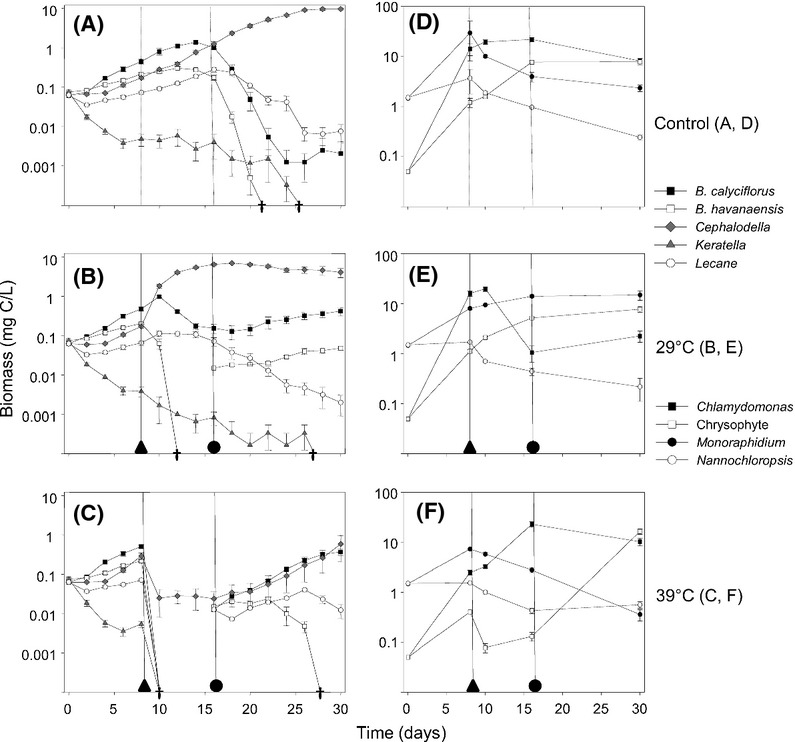
Community dynamics for the control (top), 29°C heat wave treatment (middle), and 39°C heat wave treatment (bottom), with herbivores in the left column (A, B, C, top legend box) and algae in the right column (D, E, F, bottom legend box). Heat waves started on day 8 (big black triangle) and ended on day 10. Lost species were re-introduced on day 16 (big black circle). Mean biomass (± SE) are expressed in mg C L^−1^. Crosses represent extinctions.

All phytoplankton and herbivore species, except *Keratella*, showed positive population growth and were thus considered as viable species, which in principle could take their place within the community. Only *Keratella* declined by more than an order of magnitude in the first week, in the control, and in both heat wave treatments (i.e., already before the heat wave occurred), we thus considered *Keratella* as a marginal species on its way to extinction (and it was, see the triangle-marked species decline in Fig.1A and B); it was hence not re-introduced after its more rapid extinction in the extreme heat wave treatment (as re-introduction evaluation is only useful for species that can in principle exist in the community).

In the period “before,” the heat wave populations of the different herbivore species developed similarly in the replicates of all three treatments (Fig.1A–C). Community dynamics clearly diverged between the three treatments in the heat wave period (Fig.1A–C). The Greenhouse–Geisser-corrected repeated-measures MANOVA showed a significant effect of the factor time period (*F* = 298.8, df = 1.32, *P* < 0.0001) and a significant effect of the interaction between time period and treatment (*F* = 155.7, df = 2.76, *P* < 0.0001). The associated test of within-subjects contrasts showed for factor period a significant difference between Level 1 and Later (i.e., before and after the heat wave, *F* = 779.7, df = 1, *P* < 0.0001), and showed, similarly, for the interaction between time period and treatment, a significant difference between Level 1 and Later (i.e., before and after the heat wave, *F* = 408.4, df = 2, *P* < 0.0001). These results confirm the visually clear differences in patterns for the pre- and postheat wave time periods in the different heat wave intensity treatments in Figure1.

In the period “before” the heat wave, also the populations of the different algal species developed similarly in the replicates of all three treatments (Fig.1D–F). Community dynamics diverged somewhat between the three treatments in the heat wave period (Fig.1D–F), but the difference was much less spectacular than the divergence we observed among herbivores. Divergence between the treatments was most clear during the last period of the experiment (Fig.1D–F). The Greenhouse–Geisser-corrected repeated-measures MANOVA showed a significant effect of the factor time period (*F* = 12.40, df = 1.86, *P* < 0.001, and a significant effect of the interaction between time period and treatment (*F* = 15.49, df = 3.72, *P* < 0.001. The associated test of within-subjects contrasts showed for factor period a significant difference between Level 1 and Later (i.e., before and after the heat wave, *F* = 127.41, df = 1, *P* < 0.001), and showed, similarly, for the interaction between time period and treatment, a significant difference between Level 1 and Later (i.e., before and after the heat wave, *F* = 328.27, df = 2, *P* < 0.001). So, the algal communities also changed following the heat waves and differed between heat wave intensities, supporting H1. However, algae never went extinct in our experiment.

In contrast, the most striking difference between treatments for the animals was in fact the number of extinctions of viable herbivore species. None of these went extinct in *any* of the control replicates. A single viable species, *B. havanaensis*, went extinct in *all* replicates of the 29°C treatment. In contrast, three viable species, *B. havanaensis*, *B. calyciflorus,* and *Lecane* went extinct in *all* replicates of the 39°C treatment. *Keratella* was also lost in all replicates experiencing the 39°C heat wave. The highly consistent difference in the number of herbivore species extinctions between the two heat wave intensities was significant (MWU Test, *P* < 0.0001). This strongly supports Hypothesis 1. The results clearly show different community and extinction dynamics between the heat wave intensity treatments, with the most intense extreme event leading to the highest number of extinctions.

### Differences in re-introduction success following species loss at 29 versus 39°C (H2)

*Brachionus havanaensis* successfully re-established itself in *all* replicates after the 29°C heat wave. Re-introductions of the herbivores *B. calyciflorus*, *B. havanaensis,* and *Lecane* in the post-39°C heat wave community initially all seemed successful, but *B. havanaensis*, after maintaining a relatively stable level for 6 days, rapidly declined around day 25 and went extinct in *all* post-39°C heat wave replicates. We call this “delayed community closure”. In other words, *B. havanaensis* was unable to eventually establish itself in the post-39°C heat wave community, in contrast with the post-29°C heat wave community, where it always re-established itself. This difference in re-introduction success was significant (MWU test, *P* < 0.0001). This strongly supports Hypothesis 2.

### Variation in relative abundances during reassembly post-29 versus post-39°C (H3)

**Figure 2:**
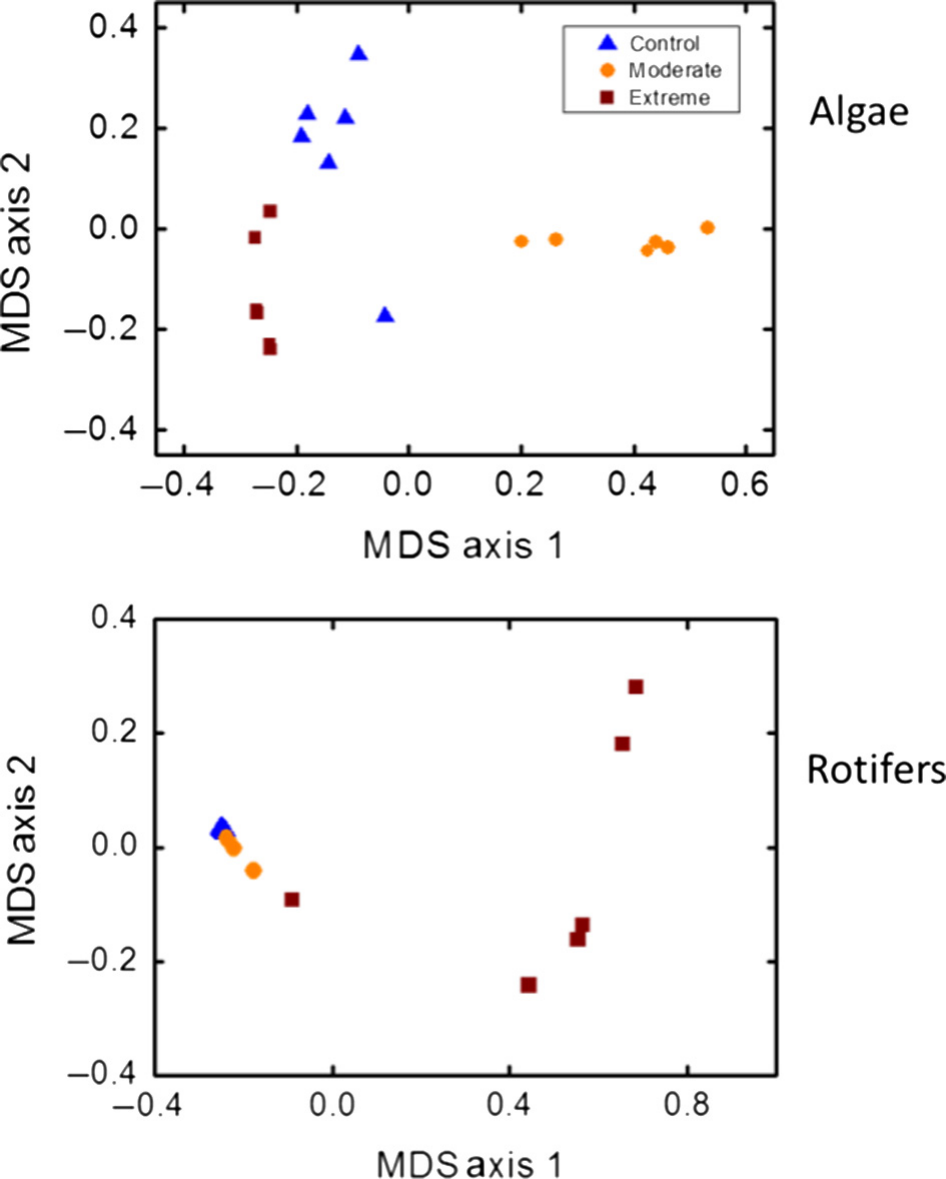
Multidimensional scaling (MDS) plot, visualizing the variability in population densities among replicates within and among treatments (control, moderate heat wave, extreme heat wave) for the final (postspecies re-introduction) period of the experiment. Variability of algae is depicted in the upper panel, of herbivores in the lower panel.

The animal communities in the post-29°C heat wave community were rather similar among replicates at the end of the experiment. In contrast, they were more variable at the end of the experiment among replicates in the post-39°C heat wave community, as indicated by the Morisita similarity index. The Morisita index ranged from 0.76 to 1.09 (median: 1.03) for the post-29°C heat wave community, whereas it ranged from 0.17 to 0.66 (median: 0.34) for the post-39°C heat wave community, at the end of the experiment. Replicates of the post-39°C heat wave animal community were significantly less similar, that is, significantly more variable, than those of the post-29°C heat wave community (*P* < 0.000001, Mann–Whitney *U*-test, at a Bonferroni-corrected critical value of 0.0033). This pattern was also visualized, now for the entire post-re-introduction period, in an MDS plot. Replicates from the “extreme” community (i.e., during *reassembly* following species introductions, after extinctions due to the 39°C heat wave) of herbivores clearly showed larger scatter than the “moderate” community, (Fig.2, bottom panel). In concert, these results for the herbivore community support Hypothesis 3. However, no increase in variability was observed for the algal community, during the last period of the experiment. Of course, community development was a different process for the algae, as none of the phytoplankton species went extinct and hence no species re-introductions took place. The phytoplankton community was thus not “reassembling” and H3 hence only applies to the herbivores (see Discussion). Graphs showing all single replicates of animal and algal communities are available as Online Supporting Information (Figs. S1–S4).

## Discussion

Recent climate change has seen an increasing frequency and intensity of extreme temperature events, including a new category of “extremely hot outliers” (Hansen et al. 2012). Projections of future climate extremes suggest that this trend will likely continue for a large fraction of the global land surface, although local trend variability is expected to be large, leading to great differences in the direction and nature of extremes among locations (Fischer et al. 2013). These extremely hot outliers may become increasingly ecologically important, as they could substantially increase local mortality among a range of species. However, such extremes in abiotic conditions could also have effects beyond inflicting direct mortality. They may fundamentally upset and change the *biotic* conditions that are essentially required for species persistence in the environment. This way an extreme event could leave an ecological legacy through changed conditions for life, even after the event itself is over. Preliminary mathematical food web model analyses confirm what is common sense and suggest that these conditions for life include not only sufficient resource availability but also survivable balances of competition and predation, next to abiotic conditions within species tolerance limits (M. Vos et al., unpubl. model analyses not shown).

The experimental work we discuss here zooms in on such “hot outliers”. We consider both moderate and more intense extreme event intensity. In particular, we discuss the recovery of herbivorous freshwater plankton communities to which lost species were re-introduced following experimental heat waves of 29°C and 39°C.

### Responses to differences in extreme event intensity

Our experimental heat waves did act as extreme events that led to animal species extinctions. The absence of extinctions in the control from days 8 to 14 shows that the extinctions occurring during that period in the two treatments were really caused by the heat wave. Under the 29°C heat wave, one of the established herbivore species, *B. havanaensis*, was lost. It went extinct in all replicates. Conversely, in the 39°C treatment, three of the four stably established herbivore species went extinct, also in all replicates. These extinctions included not only *B. havanaensis*, but also *B. calyciflorus* and *Lecane*. *Cephalodella* was a highly robust species as it survived both heat wave intensities. Nonetheless, it was strongly inhibited by the 39°C heat wave. The relatively short duration of the heat wave may have saved this species, preventing a full decline to actual extinction. *Cephalodella* recovered as soon as the heat stress was lifted. Its larger tolerance thus acted to “buy time”.

Following the extreme event, the relative abundances of the remaining animal and algal species differed between heat wave intensities (Fig.1). At the primary producer level, the 29°C treatment stimulated growth of *Monoraphidium*, at the expense of all other phytoplankton. This species became dominant. In contrast, no phytoplankton species developed such a degree of dominance in the 39°C treatment. Our results strongly support hypothesis (1) that differences in heat wave intensity lead to differences in community dynamics including numbers of extinctions and relative abundances of the remaining species.

### Trajectories of community reassembly

Species re-introductions of the lost herbivore species ensured that all viable species that had been present before the heat wave had a chance to establish themselves in the postheat wave community. However, the different heat wave intensities had also led to significant shifts in relative algal abundances between the two heat wave treatments (Fig.1E and F). These differences in resource availability in the postheat wave community can be viewed as an ecological legacy. The observed differences in relative abundance among the different phytoplankton species may have acted in concert with species-specific differences in edibility of these algae for the different herbivore species to result in different food environments for re-introduced herbivores in the postheat wave communities.

We speculate that these changes at the primary producer level are partly responsible for different outcomes in herbivore community reassembly between the different heat wave intensity treatments. These heat wave-mediated shifts in algal resources may have changed the competitive situation within the herbivore community, eventually leading to 100% re-invasion success of *B. havanaensis* in the post-29°C heat wave community and to 100% failure to re-establish in the post-39°C heat wave community. Such community closure under the highest intensity extreme event supports Hypothesis 2. Whether the observed community closure is a long-term phenomenon cannot be inferred from our experiment. Short-term disturbances can in principle lead to long-term changes in community composition, with serious implications for recovery success in natural ecosystems (Sorte et al. 2010; Eggers et al. 2012).

### Extreme event intensity and variability of recovery

As hypothesized (H1, 2, 3), we found that the 39°C high-intensity extreme event led to a higher number of animal extinctions, to community closure for one of these species, and then to a higher variability of relative animal abundances during the recovery phase, as indicated by the Morisita similarity index and as visualized in the MDS plots. We considered variability at the primary producer level as an explanatory mechanism, but found a high similarity among the post-39°C final period replicates for algal abundances. We suggest that differences among replicates in the density of the surviving *Cephalodella* may have caused part of the variability in this treatment, as these could modulate the relative success of re-introduced competing herbivores. We thus hypothesize that recovery trajectories of these re-introduced herbivores were at least in part (1) governed by algal resource availability, and (2) modulated by differences in density of the competing herbivore that still existed at varying densities in the system after the heat wave. We intend to test this in manipulative follow-up experiments.

In general, we expect a moderate heat wave to lead to fewer local extinctions than will be caused by a more intense heat wave. A smaller number of extinctions would allow a community to stay closer to its preexisting regime of ecological forces. This perspective is in line with the view that strong ecological forces tend to canalize patterns and hence decrease variability. Conversely, their disruption by (a large number of) local extinctions could enhance noise and magnify initially small differences among population densities. Our experimental results show that the consequences of a high-intensity extreme event (canalization vs. noise enhancement) may differ for different community-level phenomena. On the one hand, the most intense heat wave led to a highly repeatable number of herbivore extinctions (the same in all replicates) and to a highly repeatable community closure for one of these herbivores (also identical in all replicates). But on the other hand, the most intense heat wave led to a significantly higher variability of relative species abundances in the postre-introduction period. Community-level consequences of a high-intensity extreme event can thus seemingly include both elements of canalization and noise enhancement. We note that the latter only occurred in the herbivore community, which reassembled by means of species re-introductions, whereas it was absent in the algal community, where all species had continued to exist regardless of treatment.

### Implications for natural ecosystems

Humankind strongly relies on resilient ecological processes and a reliable provision of ecosystem services in natural, rural, and urban landscapes. This all depends on the response traits (such as heat tolerance) of the involved organisms that through their effect traits contribute to these ecosystem functions and services. The importance of resilience and reliability implies the need for a better understanding of processes underlying ecological recovery and for appropriate management strategies to support natural restoration. Our study used controlled and replicated laboratory communities as a model system, that is, a simplification of reality. Such simplification is nonetheless useful in pointing to possible processes governing recovery from high-intensity extreme events. We conclude that high-temperature extremes can on the one hand generate strong ecological processes that deterministically cause extinctions and community closure, and on the other hand generate ecological legacies that increase variability and that make the details of subsequent recovery more difficult to predict.

## Supporting Information

**Figure S1.** Control rotifer communities at 15°C. **Figure S2.** Rotifer communities of the 29°C heat wave treatment. **Figure S3.** Rotifer communities of the 39°C heat wave treatment. **Figure S4.** Algal communities of (top) the control, (middle) the 29°C heat wave and the (bottom) 39°C heat wave treatment.
