# Supplementary material for: Extreme heat changes post-heat wave community reassembly
Source: Ecol Evol. 2015 May 6;5(11):2140–8. doi: 10.1002/ece3.1490 (PMC4461416; doi:10.1002/ece3.1490)
Supplement: Supplementary file 1 [file ece30005-2140-sd1.pdf]

Extreme heat changes post-heatwave community reassembly

Running head: Extreme heat alters community recovery

Linda I. Seifert[1*], Guntram Weithoff[1], and Matthijs Vos[2, 3]

[1]Department of Ecology and Ecosystem Modelling, Potsdam University, Maulbeerallee 2, 14469 Potsdam, Germany

[2] Department of Conservation Biology, Institute of Environmental Sciences (CML), Leiden University, PO Box 9518, 2300 RA Leiden, the Netherlands

[3] Working Group Zoological Biodiversity, Ruhr-Universität Bochum, Universitätsstr. 150 / Gebäude ND05, D-44780 Bochum, Germany

*Corresponding author:

linda.seifert@uni-potsdam.de

Phone: +49(0)176 32094379

Fax: +49(0)331 977 1948

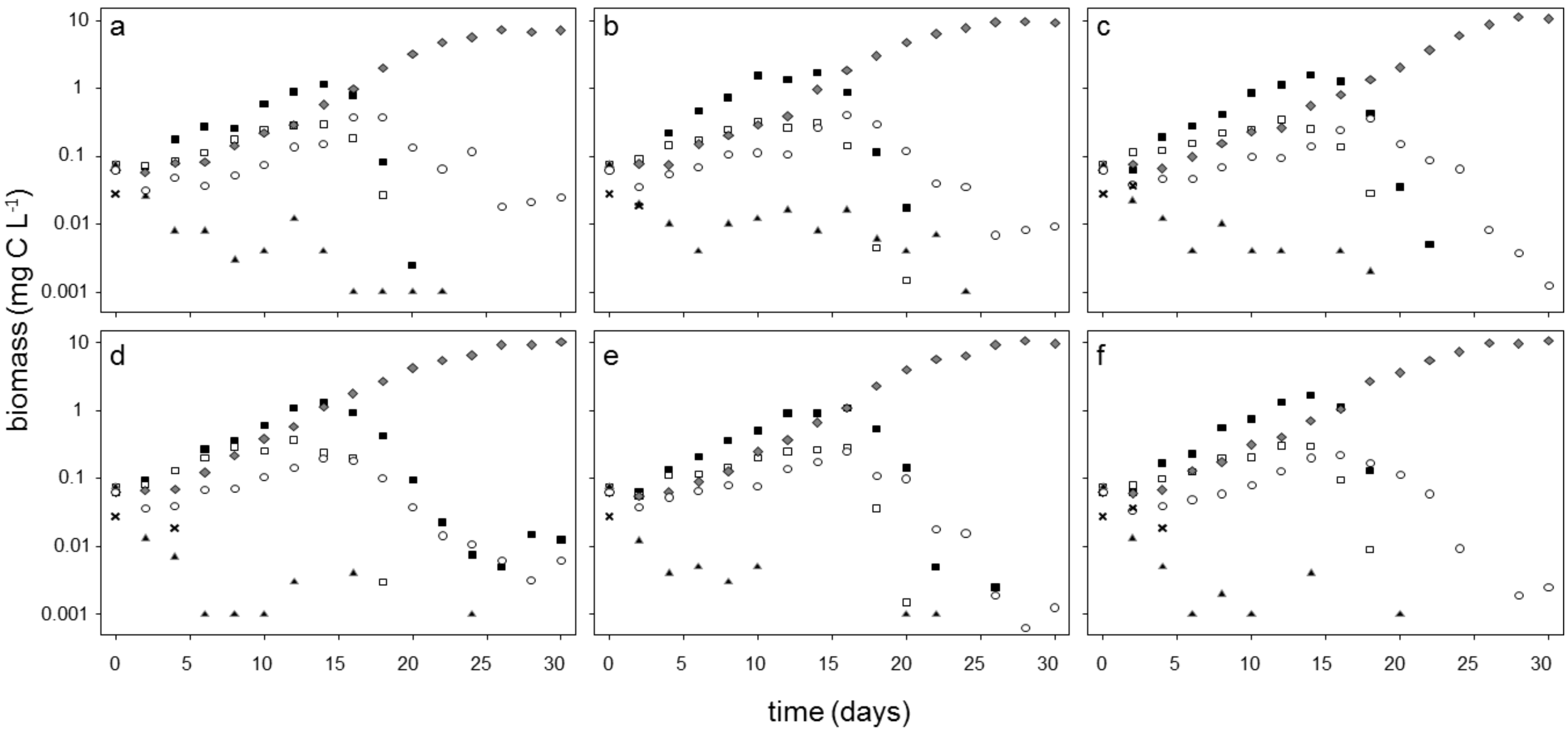

Fig. S1: Control rotifer communities at 15°C. Replicates a – f. Mean biomass (±SE) are expressed in mg C L^{-1}. Species are *Brachionus calyciflorus* (closed squares), *B. havanaensis* (open squares), *Cephalodella* (grey diamonds), *Lecane* (open circles), *Keratella* (grey triangles) and *Asplanchna* (crosses).

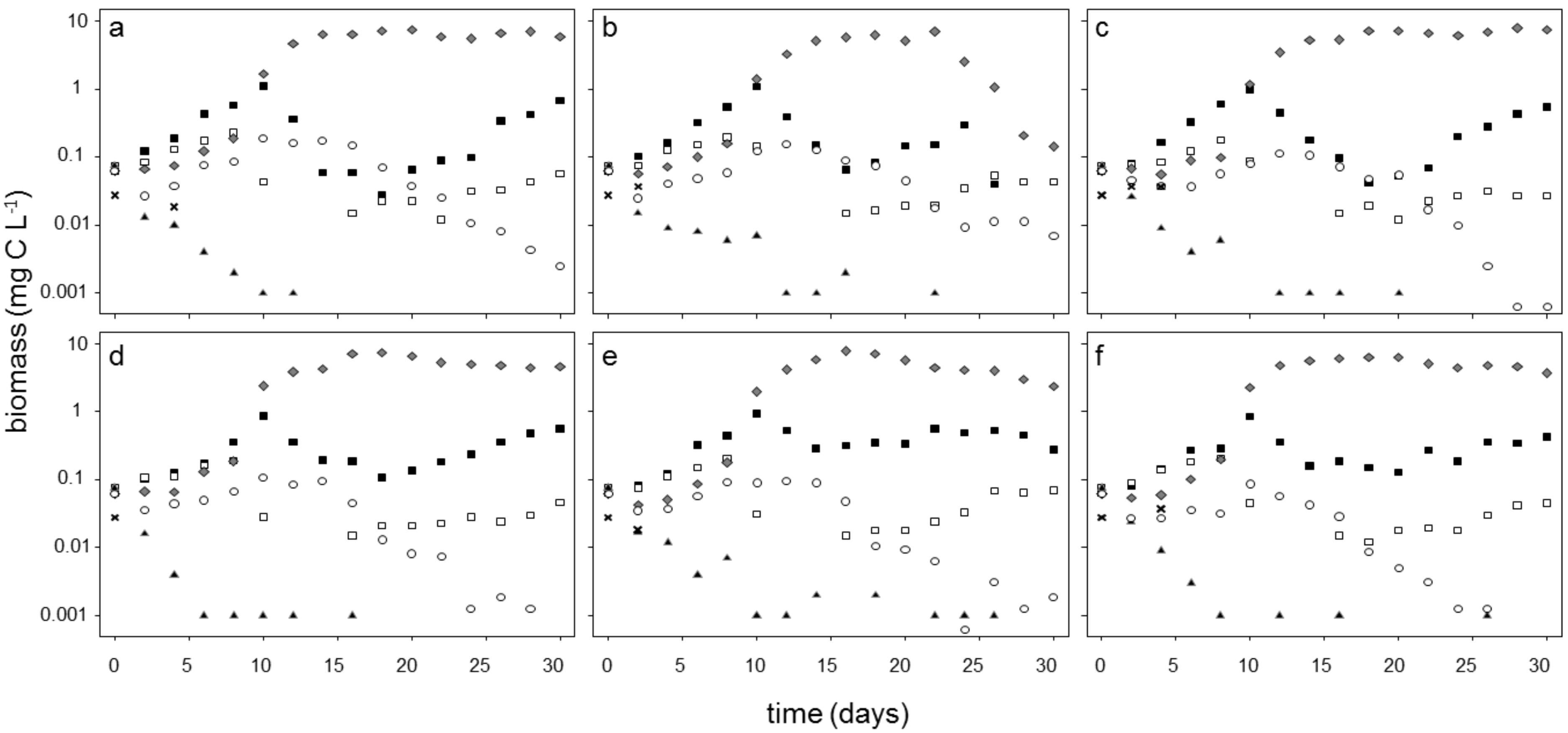

Fig. S2: Rotifer communities of the 29°C heat wave treatment. Replicates a – f. Heat waves started at day 8 and ended at day 10. Lost species were re-introduced at day 16. Mean biomass (±SE) are expressed in mg C L^{-1}. Species are *Brachionus calyciflorus* (closed squares), *B. havanaensis* (open squares), *Cephalodella* (grey diamonds), *Lecane* (open circles), *Keratella* (grey triangles) and *Asplanchna* (crosses).

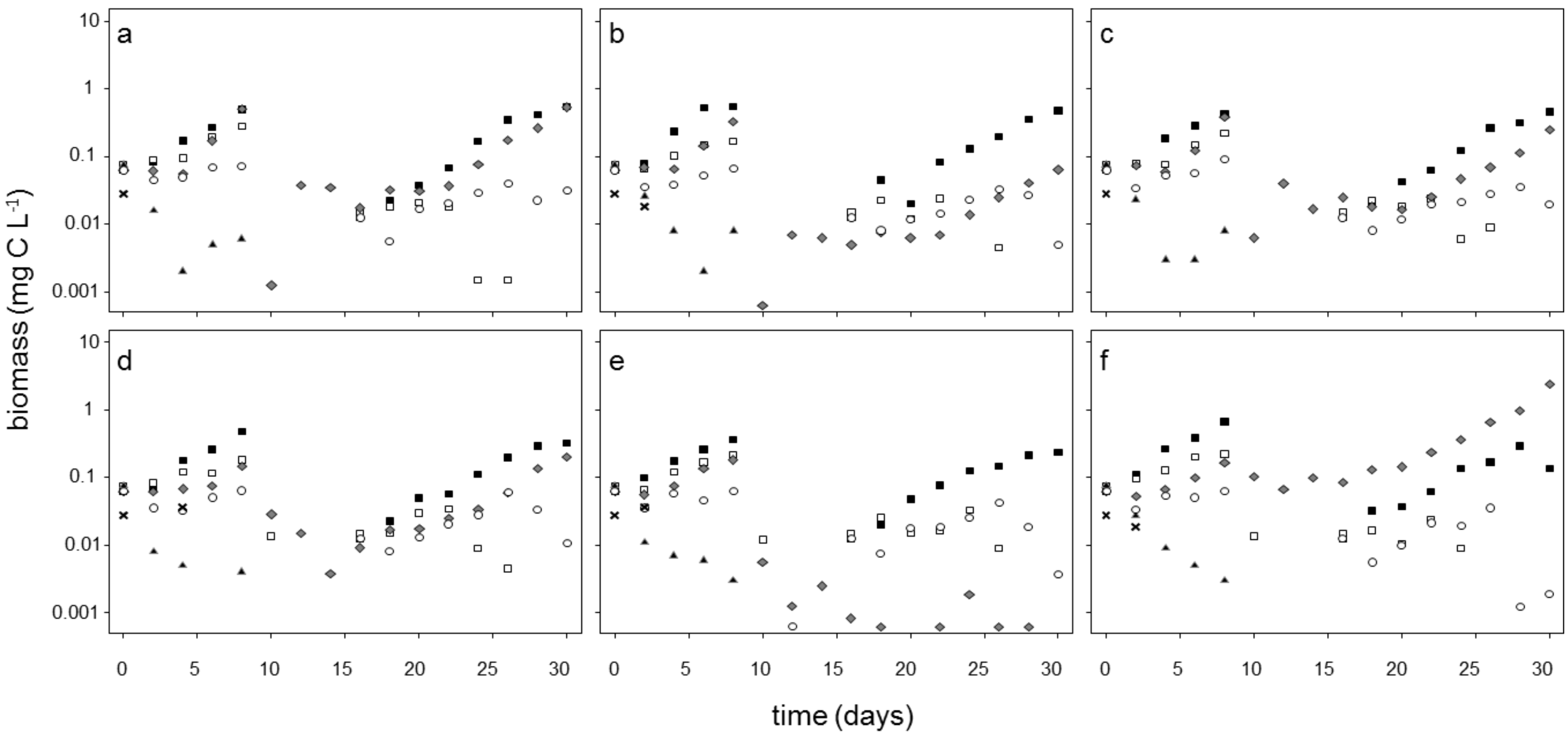

Fig. S3: Rotifer communities of the 39°C heat wave treatment. Replicates a – f. Heat waves started at day 8 and ended at day 10. Lost species were re-introduced at day 16. Mean biomass (±SE) are expressed mg C L^{-1}. Species are *Brachionus calyciflorus* (closed squares), *B. havanaensis* (open squares), *Cephalodella* (grey diamonds), *Lecane* (open circles), *Keratella* (grey triangles) and *Asplanchna* (crosses).

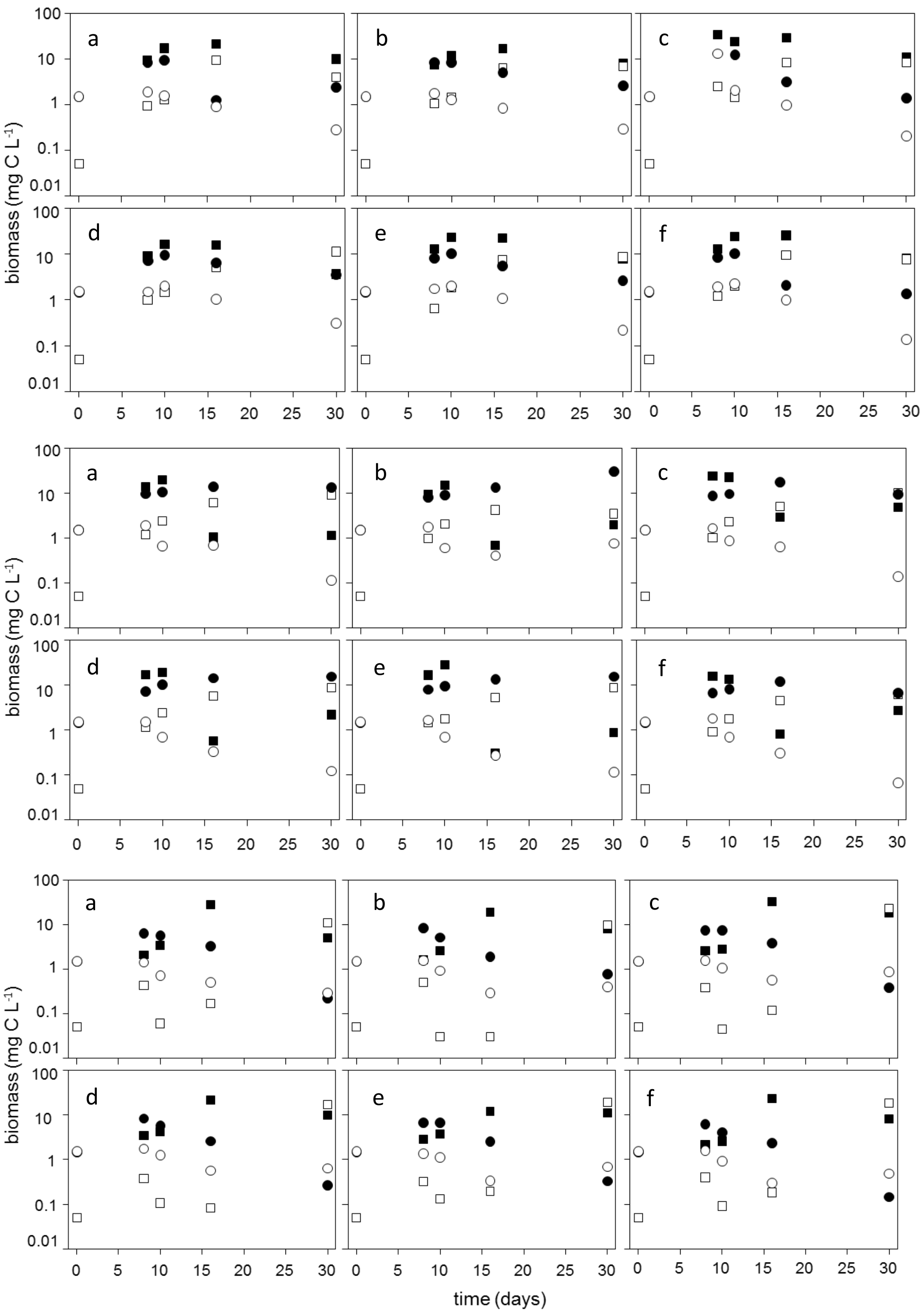

Fig. S4: Algal communities of (top) the control, (middle) the 29°C heat wave and the (bottom) 39°C heat wave treatment. Replicates a – f. Heat waves started at day 8 and ended at day 10. Species are *Monoraphidium* (closed circles), *Nannochloropsis* (open circles), *Chlamydomonas* (closed squares), Chrysophyte (open squares).
